# Lactoferrin and lactoferricin endocytosis halt *Giardia* cell growth and prevent infective cyst production

**DOI:** 10.1038/s41598-018-36563-1

**Published:** 2018-12-21

**Authors:** Lorena S. Frontera, Sofía Moyano, Gonzalo Quassollo, Adriana Lanfredi-Rangel, Andrea S. Rópolo, María C. Touz

**Affiliations:** 1 Instituto de Investigación Médica Mercedes y Martín Ferreyra, INIMEC – CONICET, Universidad Nacional de Córdoba, Córdoba, 5000 Argentina; 2 Serviço de Microscopia Eletrônica, Centro de Pesquisas Gonçalo Moniz, Fiocruz-Ba, 40296-710 Brazil

## Abstract

Lactoferrin (LF) is an 80 KDa iron-binding glycoprotein that plays a significant role in the innate immune system and is considered to be an important microbicide molecule. It has been suggested to be effective in the treatment of giardiasis, an intestinal disease caused by the protozoan parasite *G. lamblia*. However, the molecular mechanisms by which LF exerts its effect on this parasite are unknown. Most of the microbicidal activity of human or bovine LF (hLF or bLF) has been associated with the N-terminal region of the mature LF - lactoferricin (LFcin). LFcin is produced by pepsin cleavage of the native protein *in vitro* and likely *in vivo*. In this work, we analyse the participation of the endocytic machinery of *G. lamblia* in the internalization of bLF and bLFcin and their effects on cell homeostasis. Our results show that, when bLF or bLFcin are internalized by receptor-mediated endocytosis, cell growth stops, and morphological changes are produced in the trophozoites, which ultimately will produce immature cysts. Our findings contribute to disclose the fine mechanism by which bLF and bLFcin may function as an antigiardial molecule and why they have therapeutic potential to eradicate giardiasis.

## Introduction

*G. lamblia* is a protozoan parasite that inhabits the small intestine of humans and other vertebrates causing a disease known as giardiasis. Globally, ingestion of *G. lamblia* cysts, present in water or contaminated food, is the most common cause of diarrheal disease not associated with viruses or bacteria, and may affect individuals with a normal as well as with an altered immune system. In the environment, *G. lamblia* is protected by an outer shell (cyst wall) that allows it to survive outside the host for long periods of time and makes it tolerant to hypochlorite disinfection^1,2^. Inside the host, the cysts become trophozoites that colonize the intestine and cause the symptoms of the disease. As a result of infection, the body’s ability to absorb fat, lactose, vitamin A, and vitamin B12 is commonly affected and this may lead to weight loss and can cause malnutrition, especially in children. Only a handful of drugs are in use for the treatment of pathogenic protozoan parasites and, in many cases, the infection is inevitably fatal without medical intervention^3,4^. Given that vaccination is not an option for all pathogens and that the chronic use of microbicide agents produces resistant strains, there is a critical need for the discovery of natural microbicides, as is the case of lactoferrin (LF) and its products. However, this cannot be achieved without a better understanding of the basic biology of the parasite, in particular of the processes that may be susceptible to therapeutics.

A large amount of research has focused on the structure and function of the iron-binding protein LF, achieving considerable advances in our understanding of its synthesis, distribution and degradation. It is now recognized as a molecule with multiple biological roles, including regulation of iron absorption, and as a protein with anti-oxidant, anti-microbial, anti-carcinogenic and anti-inflammatory functions, among others recently discovered^5^. Human and bovine milk are the most abundant source of LF but it is also present in saliva, seminal fluid, glandular epithelial cells, and neutrophils^6,7^. It was shown that the degradation of LF to its active peptides, lactoferricin (LFcin) and lactoferrampin, produced during digestion^8,9^, may be advantageous since the anti-microbial effect of these peptides was proved *in vitro* to be more potent against microorganisms than the native protein^10,11^. However, it is now known that LF degradation depends not only on its degree of glycosylation (specie-specific) and iron saturation but also on the method of administration, intragastric pH, and type and activity of gastric and intestinal enzymes, which are highly variable between adults and infants^12^. Although more and more functions are being discovered, the mechanism of the entry of LF (and its peptides) to the cell is not completely clear. New candidates involved in this uptake have been postulated. It has been suggested that the multiple biological activities of LF depend on its target cells and on the presence of specific receptors (LfR) on their surface^13–15^. However, recent evidence showed the participation of the low-density lipoprotein receptor-related protein (LRP) in Lf endocytosis by several cell types^16–18^. This new finding prompted us to analyse whether, in the parasite *G. lamblia*, an LRP-like receptor is involved in the internalization of LF, since no LfR has been found in this parasite but a receptor-mediated endocytosis (RME) mechanisms may be involved^19–21^. On the one hand, Turchany *et al*. suggested that either human or bovine lactoferrin (as well as their LFcin derivatives) internalization was mediated by an unknown receptor and produced irreversible cell damage in the ultrastructure of this parasite^19^. On the other, we reported a few years ago that *G. lamblia* possesses a RME mechanism for the endocytosis of low-density lipoprotein (LDL) and chylomicrons via their binding to the giardial GlLRP receptor (GL50803_113565)^22–24^. Thus, it is possible that, in the absence of a bona fide LfR orthologue in the *Giardia* genome database (GiardiaDB http://giardiadb.org/giardiadb/), GlLRP is involved in the endocytosis of LF and its derived peptides in *G. lamblia*.

To analyse whether the internalization of LF could be mediated by RME, we used different strategies to interfere in LF entry at different levels of the endocytosis process. Our hypothesis is that LF enters the trophozoite through the GlLRP receptor, competing with LDL and chylomicrons for the receptor and the endocytic machinery. The results display the role of the RME machinery in the regulated endocytosis of bLF and bLFcin and the initiation of cell damage that induces cell differentiation but blocks encystation to infective-mature cysts. These findings reinforce the use of these molecules as natural killers for giardiasis treatment.

## Results

### bLF and bLFcin bind to a surface protein receptor

**Figure 1:**
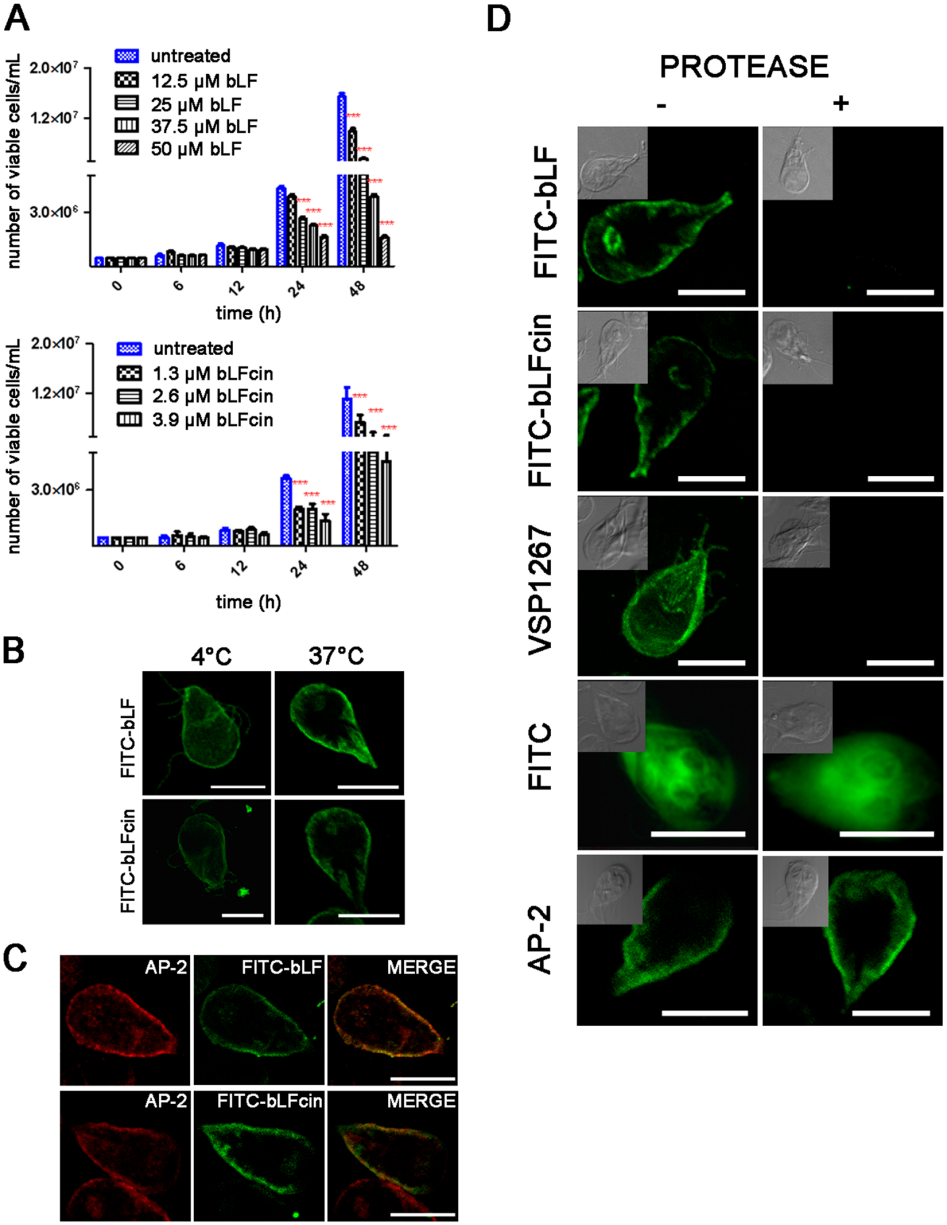
bLF and bLFcin bind to a protein receptor. (**A**) Growth curves of *wild-type* trophozoites (WB/1267) with different concentrations of bLF and bLFcin. The number of viable cells was determined by counting with a haemocytometer after addition of Trypan blue. Data shown are representative of three independent experiments and are expressed as means of triplicates ***p-value < 0.001. (**B**) FITC-bLF and FITC-bLFcin confocal microscopy shows the fluorescence on the trophozoite surface (including the flagella) in ice-cold medium (0–4 °C) but inside the cell after 30 min of incubation at 37 °C. (**C**) FITC-bLF and FITC-bLFcin (green) co-localize (yellow in MERGE) with AP-2 (red) at the peripheral vacuoles (PVs). (**D**) *wild-type* trophozoites show the localization of FITC-bLF and FITC-bLFcin in the PVs (−), beneath the plasma membrane (left panels) but, after protease treatment (+), neither FITC-bLF nor FITC-bLFcin is seen inside the cell (right panels). The extracellular portion of VSP1267 was degraded and unseen in trophozoites treated with proteases. FITC alone was able to penetrate untreated and treated trophozoites. Protease treatment did not distort the PVs localization below the plasma membrane (AP-2). Differential interference contrast (DIC) microscopy images are shown as insets. Bar, 5 μm.

Comparison of the antimicrobial activities of human, bovine, murine, and caprine LFcin showed that bovine LFcin was the most potent^25^. In this work, we used bovine apo-LF (containing 4.1% iron) and the iron-free bovine LFcin (bLFcin) since these would be the most accessible molecules for therapeutic purposes and showed an equal rate of intake by trophozoites^19,20^. To analyse the entry mechanisms of bLF and bLFcin in healthy cells, we first performed a growth curve of *wild-type* trophozoites (strain WB/1267) using different concentrations of bLF and bLFcin, to find the lowest growth inhibitory concentration that would allow us to carry out internalization experiments without causing the death of the trophozoites. We found that concentrations of 12.5 μM and 2.6 μM of bLF and bLFcin, respectively, were sufficient to inhibit cell growth without apparent cell damage or significant killing effect, determined by the cells labelled with Trypan blue (Fig. 1A). All the further experiments were performed using these concentrations, unless specified.

It was shown that in *G. lamblia* either hLF or bLF and their peptides remained on the surface of the trophozoites when the cells were maintained at 4 °C, a condition that non-specifically inhibits receptor-mediated endocytosis^19–21^. We first confirmed that both FITC-labelled bLF and bLFcin remained on the trophozoite surface in ice-cold medium (0–4 °C) but were observed inside the cell after 30 min of incubation at 37 °C (a situation that restored endocytosis) (Fig. 1B). As shown in Fig. 1C, FITC-bLF and FITC-bLFcin were localized in the peripheral vacuoles (PVs), organelles that function as endosomes and lysosomes and are located beneath the plasma membrane, co-localizing with the PV-marker adaptor protein AP-2. Then, to reveal whether a protein receptor is involved, we performed extracellular protease digestion to eliminate the extracellular portion of membrane proteins, before testing bLF and bLFcin binding and internalization. Neither FITC-bLF nor FITC-bLFcin was bound to the membrane after protease treatment, while binding and internalization were observed for untreated cells (Fig. 1D). This treatment resulted in digestion of the extracellular portion of the variable-surface protein VSP1267 used as an assay control (Fig. 1D). These results reinforced the participation of a receptor in the endocytosis of bLF and bLFcin in *G. lamblia*. FITC alone was able to enter both untreated and treated cells, as expected (Fig. 1D). Protease treatment did not alter the PVs as can been seen by the staining of the PVs by using the anti-AP-2 mAb in post-fixed permeabilized cells (Fig. 1D).

### GlLRP participates in the endocytosis of bLF and bLFcin

**Figure 2:**
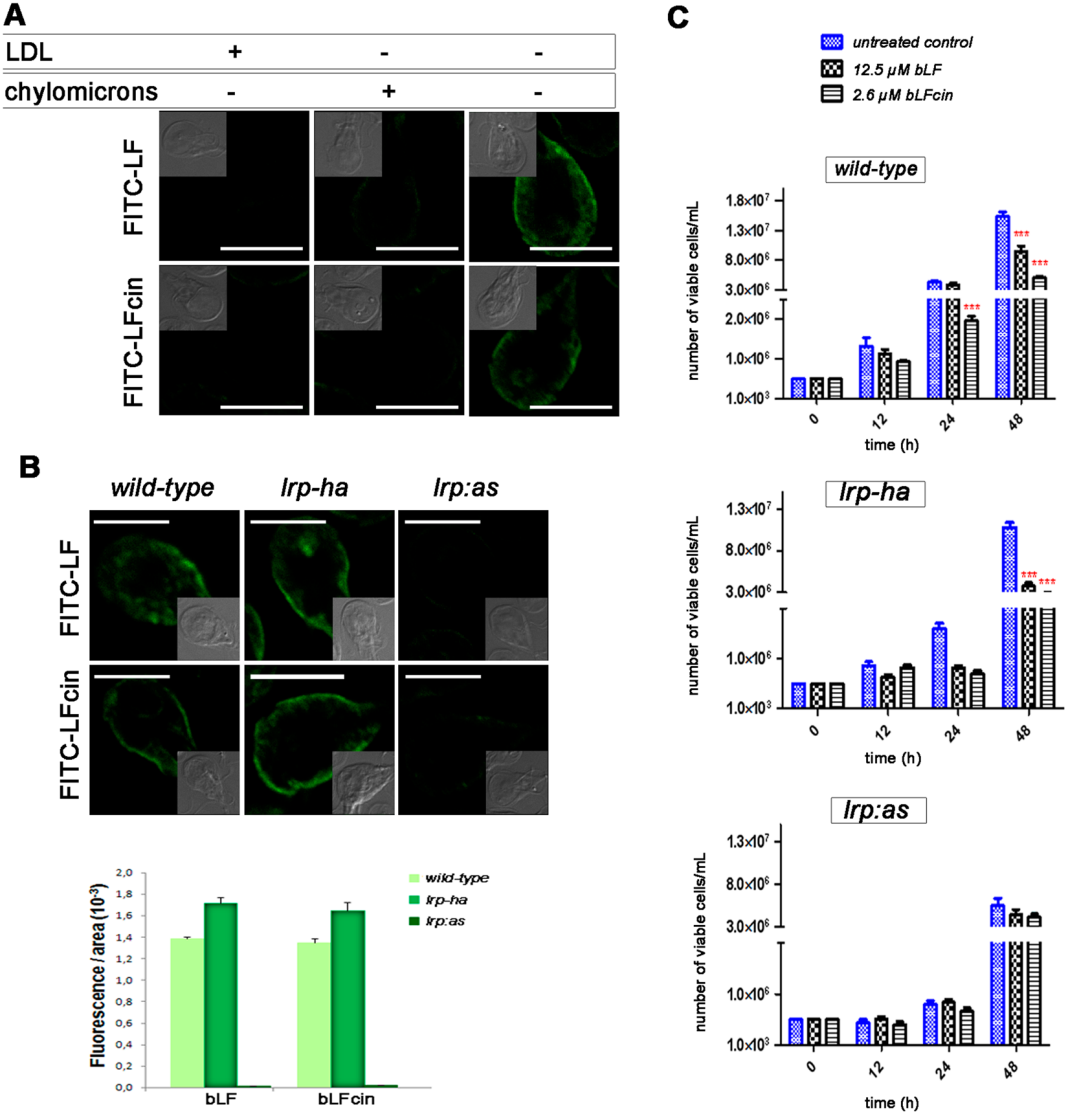
GlLRP is involved in the endocytosis of bLF and bLFcin. (**A**) *Wild-type G. lamblia* trophozoites challenged with unlabelled LDL (+) or chylomicrons (+) show a reduction of FITC-bLF and FITC-bLFcin uptake compared with unchallenged trophozoites (−). The most representative effect is shown for each treatment. (**B**) FITC-bLF and FITC-bLFcin are endocytosed to the PVs in *wild-type* and *lrp–ha* but not in *lrp:as* trophozoites. The most representative effect is shown for each type of cell. Differential interference contrast microscopy images are shown as insets. Bars, 5 μm. Histograms of fluorescent images (fluorescence/area × 10^3^) show the relative amount of fluorescent bLF or bLFcin internalized by parasites in control (*wild-type*) and transgenic cells (*lrp–ha* and *lrp:as*). All images were equally processed; the threshold value was determined and is exclusive in each image. The results are presented here as the average (±SD) of 10 determinations. (**C**) *lrp:as* transgenic trophozoites treated with 12.5 μM of bLF or 2.6 μM of bLFcin show a non-deleterious effect on cell growth compared with *wild-type* and *lrp-ha* cells. Data shown are representative of three independent experiments and are expressed as means of triplicates ***p-value < 0.001.

To analyse whether GlLRP is implicated in the endocytosis of bLF and bLFcin, we challenged *wild-type* cells with the GlLRP natural ligand LDL or chylomicrons before adding different concentrations of FITC-bLF or FITC-bLFcin in a lipoprotein-deficient medium. The results showed that the uptake of even up to 25 µM FITC-bLF or 5.2 µM FITC-bLFcin was completely inhibited when the trophozoites were previously incubated with either of the lipoproteins (Fig. 2A). Lipoprotein pre-incubation had no effect on FITC internalization (not shown). These results prompted us to determine the endocytosis of bLF and bLFcin in cells lacking GlLRP^22^ to establish a functional connection between GlLRP and these molecules. For this, we performed endocytic uptake of FITC-bLF and FITC-bLFcin in *wild-type* trophozoites (as a control), *lrp-ha* (cells overexpressing GlLRP-HA), and *lrp:as* (cells specifically-depleted of GlLRP by antisense)^22^. Before performing the experiments, the relative mRNA expression of the transfected cells was analysed as well as the impaired endocytosis of BODIPY-LDL in knock-down trophozoites (Supplementary Fig. 1A)^22^. Fluorescence microscopy showed a significant reduction of both bLF and bLFcin endocytosis in *lrp:as* compared to *lrp-ha* and *wild-type* trophozoites (Fig. 2B). Interestingly, growth curves of *lrp:as* trophozoites under bLF or bLFcin treatment showed no effect on cell growth when compared with the *lrp:as* cells without treatment, clearly suggesting that the participation of GlLRP is critical for bLF and bLFcin uptake and microstatic effects (Fig. 2C). Conversely, *lrp-ha* and *wild-type* trophozoite growth was severely affected by the addition of bLF or bLFcin (Fig. 2C). Because *lrp:as* has a marked effect on cell growth^22^, each curve was performed independently.

### The endocytic machinery is involved in bLF and bLFcin uptake

**Figure 3:**
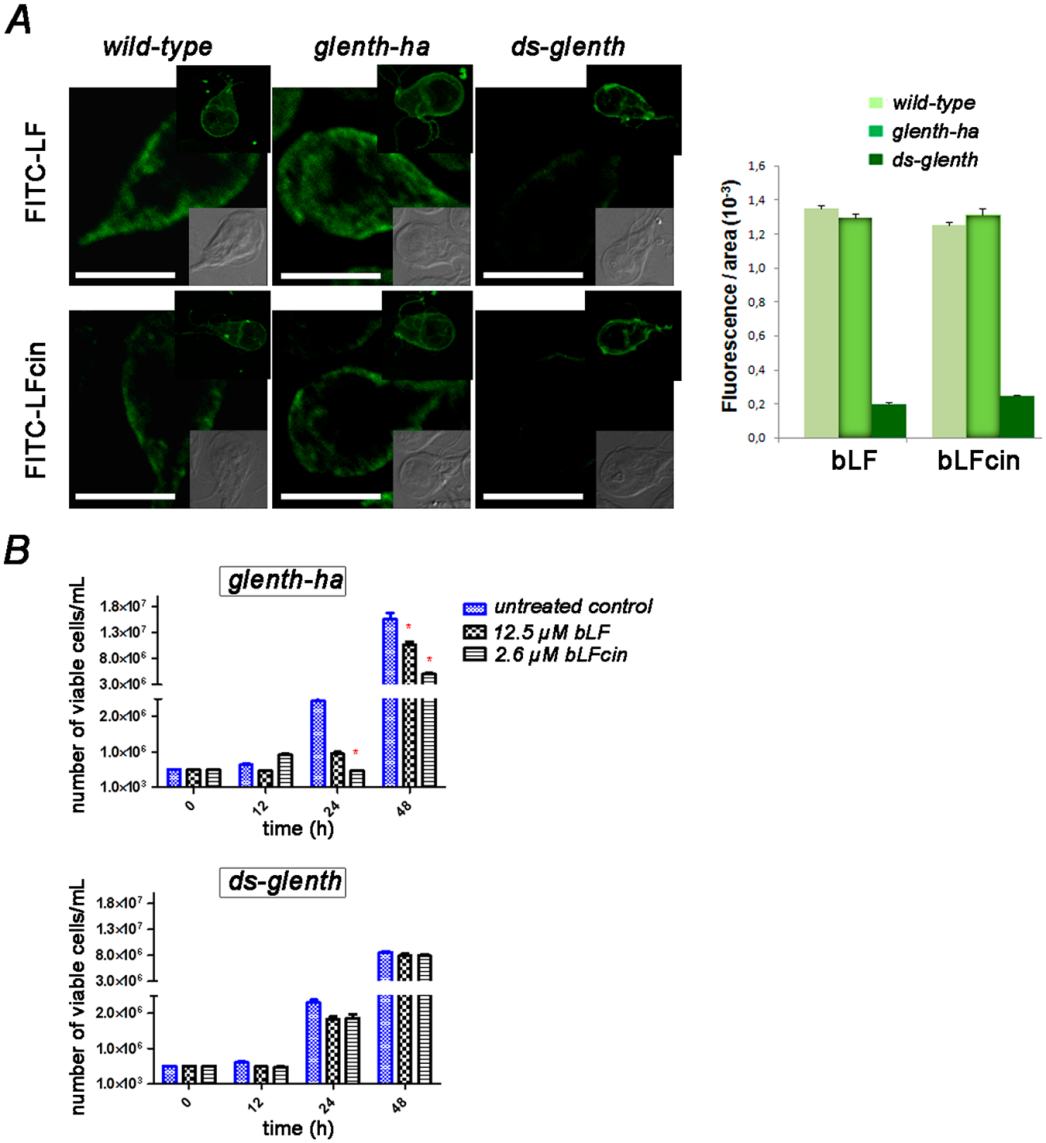
The clathrin-adaptor protein GlENTHp participates in bLF and bLFcin uptake. (**A**) FITC-bLF and FITC-bLFcin are endocytosed to the PVs in *wild-type* and *glenth–ha* but not in *ds-glenth* trophozoites. The most representative effect is shown for each type of cell. Differential interference contrast (DIC) microscopy images are shown as insets. Bars, 5 μm. Representative image of *wild-type* and transgenic trophozoites showing the binding of FITC-bLF and FITC-bLFcin on the cell surface (including the flagella) when treated 10 min at 4 °C, washed and fixed with 4% formaldehyde (top insets). Histograms of fluorescent images (fluorescence/area × 10^3^) show the relative amount of fluorescent bLF or bLFcin internalized by parasites in control (*wild-type*) and transgenic cells (*glenth–ha* and *ds-glenth*). All images were equally processed; the threshold value was determined and is exclusive in each image. The results are presented here as the average (±SD) of 10 determinations. (**B**) *ds-glenth* transgenic trophozoites treated with 12.5 μM of bLF or 2.6 μM of bLFcin show a non-deleterious effect on cell growth compared with *glenth-ha* cells. Data shown are representative of three independent experiments and are expressed as means of triplicates ***p-value < 0.001.

As a strategy to test the participation of the clathrin-adaptor proteins GlENTHp (GL50803_3256)^26^ in the internalization and PV localization of bLF and bLFcin, we took advantage of the transgenic cells, *glenth-ha* (trophozoites overexpressing the monomeric clathrin-adaptor protein GlENTHp-HA) and *ds-glenth* (double-stranded *knock-down* of GlENTHp) (Supplementary Fig. 1B)^24^. When we added FITC-bLF or FITC-bLFcin to *wild-type* or transgenic trophozoites, we observed a significantly lower internalization of these fluorescent molecules in *ds-glenth* trophozoites compared to *wild-type* or *glenth-ha* transgenic trophozoites (Fig. 3A). Binding assay showed that FITC-bLF and FITC-bLFcin were located on the surface of the trophozoites at 4 °C, indicating that the receptor-binding also occurred in these transgenic cells (Fig. 3A, top inserts). When we tested the effect of bLF and bLFcin treatment on these cells in 48 h cultures, their addition produced a marked cytostatic effect on *wild-type* (Fig. 2C) and *glenth-ha* transgenic trophozoites but not on *ds-glenth* cells (Fig. 3B), reinforcing the fact that the entry of bLF and bLFcin by RME is necessary to produce the cytotoxic effect. Similar to what occurs with *lrp:as*, the depletion of GlENTHp causes significant harm to trophozoite growth^24^ and, thus, each curve was performed independently. The toxicity effect of bLF and bLF-cin on overexpressing *lrp-ha* transgenic cells seemed to be more drastic than on the *wild-type* and *glenth-ha* cells, suggesting that more expression of the receptor lead an augment in bLF and bLF-cin internalization and toxicity. Altogether, these results show the participation of GlLRP and the endocytosis machinery in the internalization of bLF and bLFcin at low doses. As the internalization of human and bovine LF and their peptides produced strong cell damage and killing effects at high doses^19–21^, we examined whether there is any effect of these molecules at the low doses tested in this work.

### Low concentrations of bLF and bLFcin altered the homeostasis of the trophozoites

**Figure 4:**
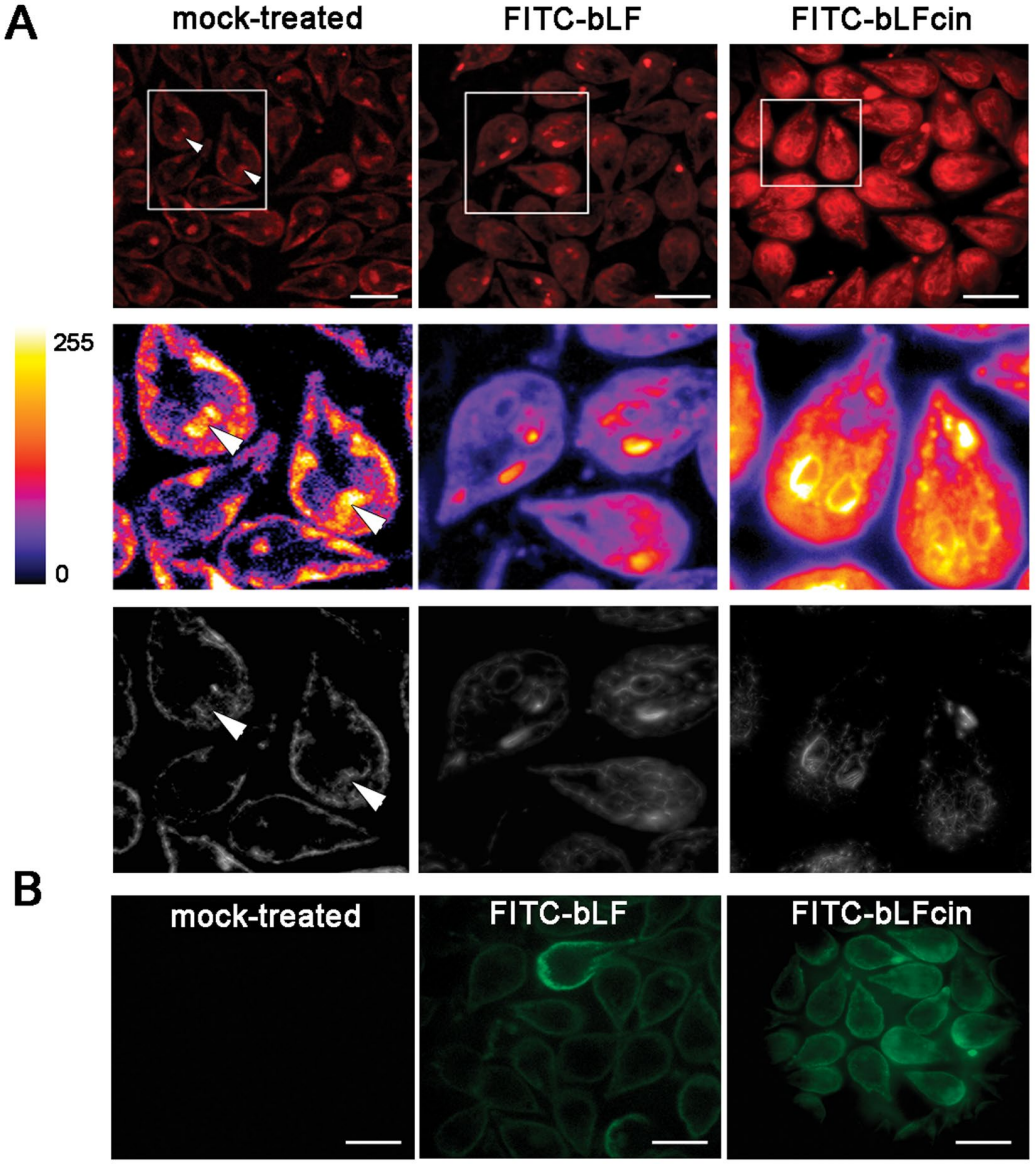
bLF and bLFcin cell treatment produce acidification of the ER. (**A**) Addition of LysoTracker (red) to w*ild-type* trophozoites treated with 12.5 μM of FITC-bLF or 2.6 μM of FITC-bLFcin shows acidification of the ER. In mock-treated w*ild-type* trophozoites, only the acidic PVs, including the bare zone (arrowhead), are labelled with LysoTracker. Insets show the staining intensity transformed into a Fire Lookup Table with the ImageJ program (colour bar), in which fluorescence intensity increases from black (0) to white (255) (middle panels) and deconvolution using Super-resolution radial fluctuations, in which the reticular network of the ER is clearly denoted in treated cells (bottom panels). (**B**) Same images showing that FITC-bLF and FITC-bLFcin are located in the PVs (green) after treatment. Bars, 10 μm.

**Figure 5:**
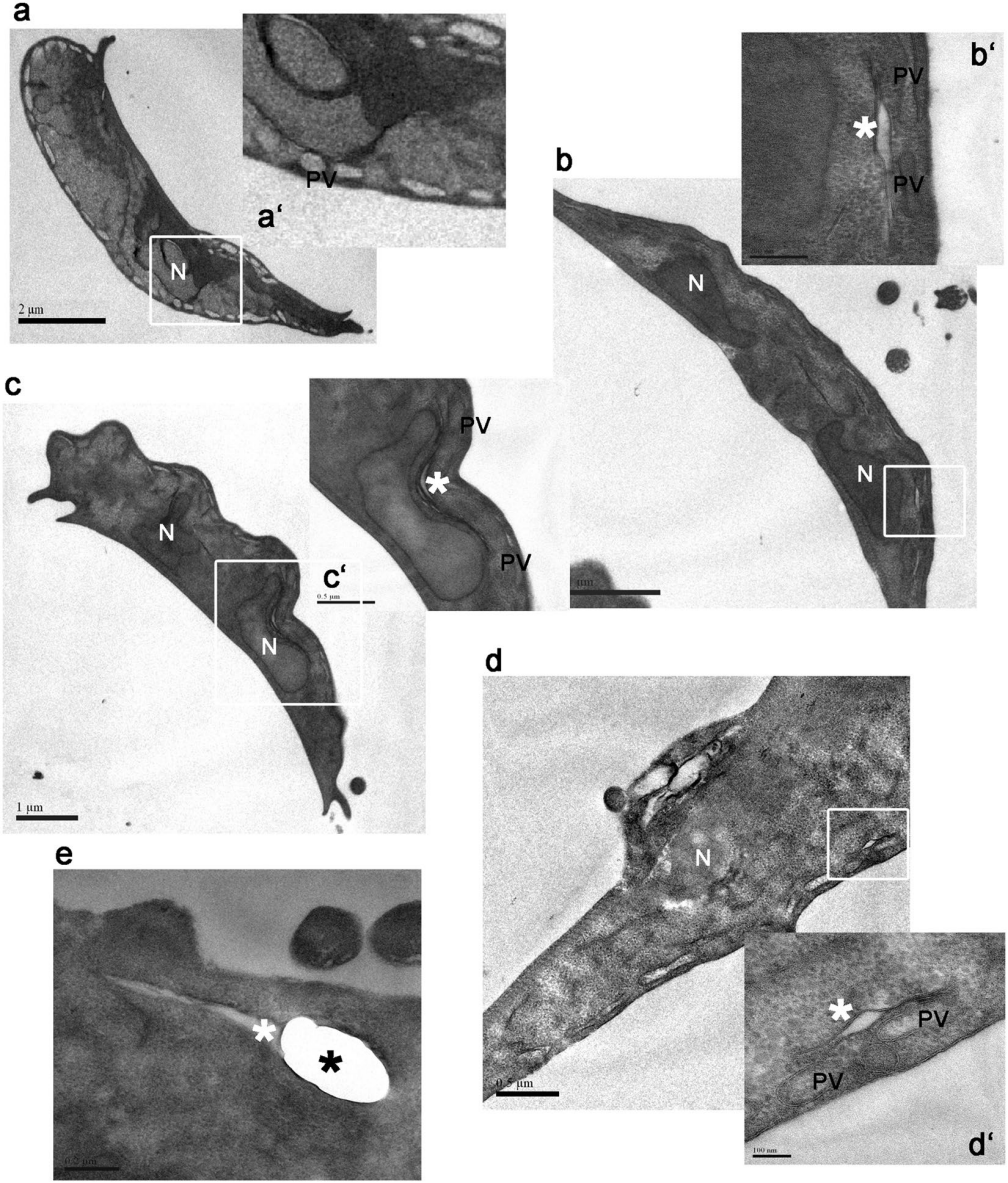
bLF and bLFcin cause morphological subcellular alterations on trophozoites. Electron microscopy shows *G. lamblia* trophozoites without addition of bLf or bLFcin (**a**) and trophozoites treated with 12.5 μM of bLF (**b,c**), or 2.6 μM of bLFcin (**d,e**). Insets show alteration of the electron-dense material of the PVs (PV), clefts formed by ER opening (white *), and nuclear membrane expansion only in treated cells (b’–d’) but not in the control (a’). A more drastic effect on the ER is observed in cells treated with bLFcin (e, black *). N: nucleus. PVs: Peripheral Vacuoles. Bar sizes are depicted in each figure.

**Figure 6:**
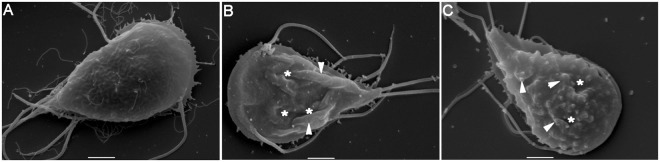
Plasma membrane deformations are caused by bLF and bLFcin at low concentrations. Scanning electron microscopy of *wild-type* trophozoites without addition of bLf or bLFcin (**A**) shows intact, smooth plasma membrane, while trophozoites treated with 12.5 μM of bLF (**B**), or with 2.6 μM of bLFcin (**C**), show membrane invaginations (*) and protrusions (arrowhead) but not holes or perforations. The most representative picture is shown for each type of cell. Bars, 2 μm.

It has been shown by Turchany *et al*. (1997) and Aguilar-Diaz *et al*. (2016) that bLF and bLFcin produce a variety of morphologic effects on treated trophozoites, ranging from changes in the cytoskeleton to endomembrane structures, including marked vesiculation of the endoplasmic reticulum (ER), enlargement of the nuclear envelope and delocalization of the PVs containing electron-dense material^19–21^. These alterations seem to be a clear effect of the start of cellular necrosis, as a cause of the high concentration of LF and LFcin used in these assays. Since no studies have been made of morphological defects using low concentrations of these molecules, we decided to analyse first whether the PVs were altered after treatment with bLF and bLFcin. The character of PVs as an acidic compartment was assessed by adding the fluorescent cell-permeant weak base LysoTracker, a molecule that specifically labels acidic compartments like the PVs and including the bare zone (Fig. 4A, mock-treated)^23,27^. Intriguingly, we found the ER acquired acidic characteristics when the cells were treated with low concentrations of bLF or bLFcin (Fig. 4A). Still, FITC-bLF and FITC-bLFcin localized inside the PVs (Fig. 4B). The proper localization of the PVs after FITC-bLF and FITC-bLFcin treatment was also assessed by using the 2F5 mAb, which detects the PV-resident protein AP-2^23^ (Fig. 1C). Thus, at low concentration, these molecules seemed not to disrupt PVs localization but rather altered the acidity of the ER by an unknown mechanism. To analyse this further, we prepared the trophozoites for transmission and scanning electron-microscopy (TEM and SEM). Under the same treatment, the trophozoites showed more electron-dense PVs and the appearance of dilated ER membranes forming clefts (Fig. 5). When treated trophozoites were observed under SEM, they exhibited plasma membrane invaginations and protrusions with no perforations or holes observed on the membrane, compared with untreated trophozoites (Fig. 6). These observations showed that, at low concentrations, bLF and bLFcin did not produce severe morphological alterations of the trophozoites. However, they caused dilation of the ER membranes (clefts), expansion of the nuclear membrane and plasma membrane protrusions, events also described during the first step of cell differentiation into cysts^28–30^.

### bLF and bLFcin activate cell differentiation

**Figure 7:**
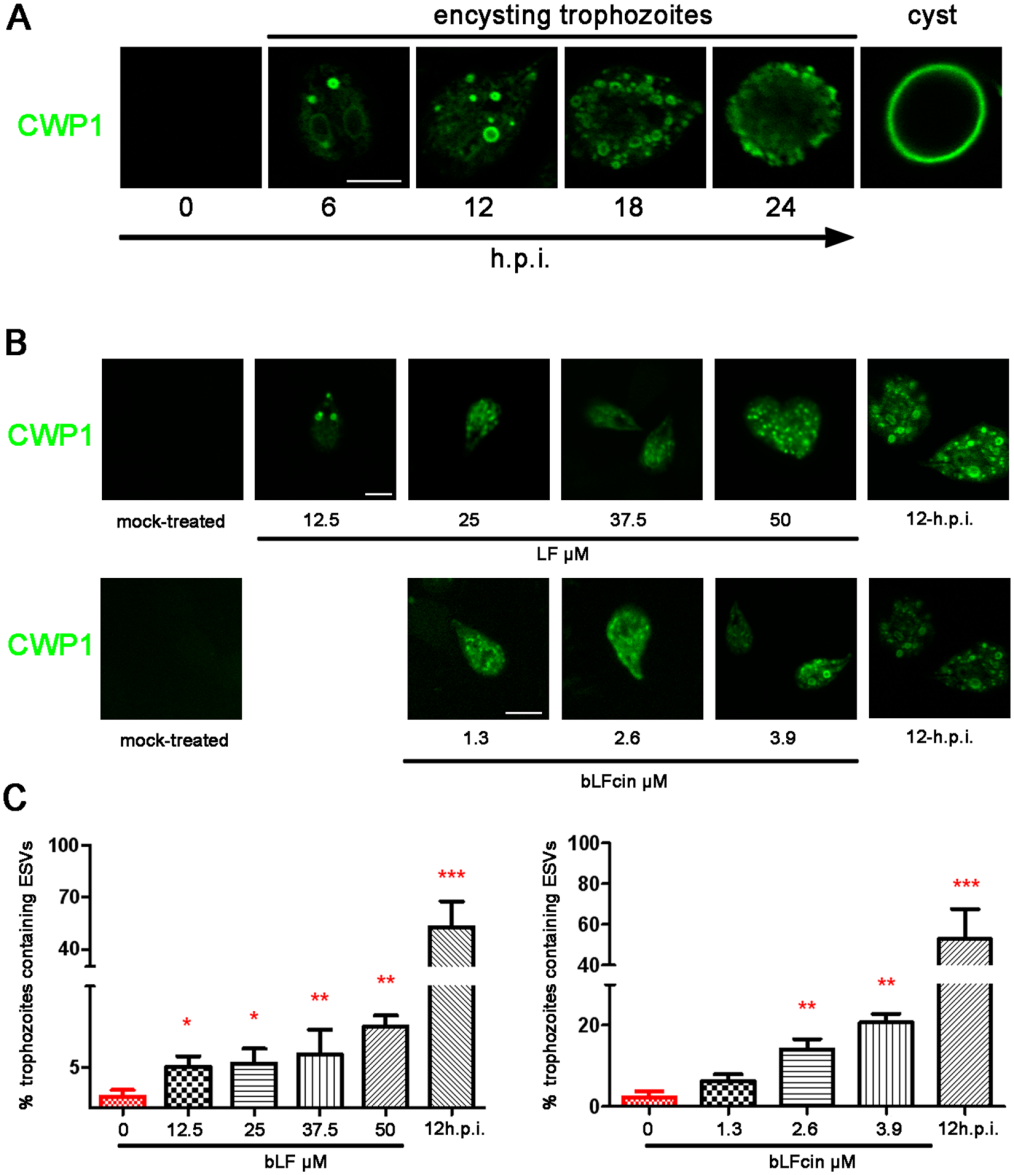
Addition of bLF and bLFcin to growing *wild-type* trophozoites induces CWP1 expression and ESVs formation. (**A**) The encystation process: Immunofluorescence assays and confocal microscopy show that CWP1 (in green) is not expressed on non-encysting trophozoites but appeared in *de novo* formed ESVs after 6 hours post-induction (h.p.i.) of encystation. Finally, CWP1 is released to constitute the cyst wall (cyst). Bar, 5 μm. (**B**) After treatment, CWP1 is observed in trophozoites exposed to different concentrations bLF or bLFcin in Labeling buffer lacking serum for 30 min. No CWP1 signal is detected in mock-treated growing trophozoites. Trophozoites encysting for 12 h (12 h.p.i) are shown as control of the IFA. Bar, 5 μm. (**C**) The graphics show the percentage of trophozoites with formed ESVs which increases as we raise the dose of bLF or bLFcin. Data shown are representative of three independent experiments and are expressed as means of triplicates ***p-value < 0.001, **p-value < 0.01, and *p-value < 0.05.

We wondered whether low doses of bLF and bLFcin might mimic the trigger that induces the differentiation to cyst. Encystation begins in response to changes in the trophozoite environment, including increased bile concentrations, alkaline pH and depletion of cholesterol^31^. As a consequence, the proteins that will form the cyst wall (CWP1-3, cyst wall proteins 1 to 3) are specifically synthesized and transported into encystation-specific vesicles (ESVs) to reach the plasma membrane (Fig. 7A). Similarly, the addition of bLF or bLFcin to growing trophozoites, maintained in Labeling buffer lacking serum for 30 min, washed with the same medium without bLF or bLFcin at 4 °C and immediately fixed using 4% formaldehyde, promoted the expression of CWP1 and the formation of ESVs. Moreover, we observed that the number of trophozoites containing ESVs increased with the addition of increased amounts of bLF or bLFcin (Fig. 7B). Mock-treated trophozoites processed equally did not show CWP1 expression or ESVs formation, suggesting that treatment with bLF or bLFcin is responsible for CWP1 expression (Fig. 7B, mock-treated). No cysts-like cells were observed at any treatment concentration and these trophozoites remained as encysting-like cells for more than 24 h (not shown). Trophozoites encysting for 12 h (12 h.p.i) were tested as control of the immunofluorescence assay.

### bLF and bLFcin produce futile cysts

**Figure 8:**
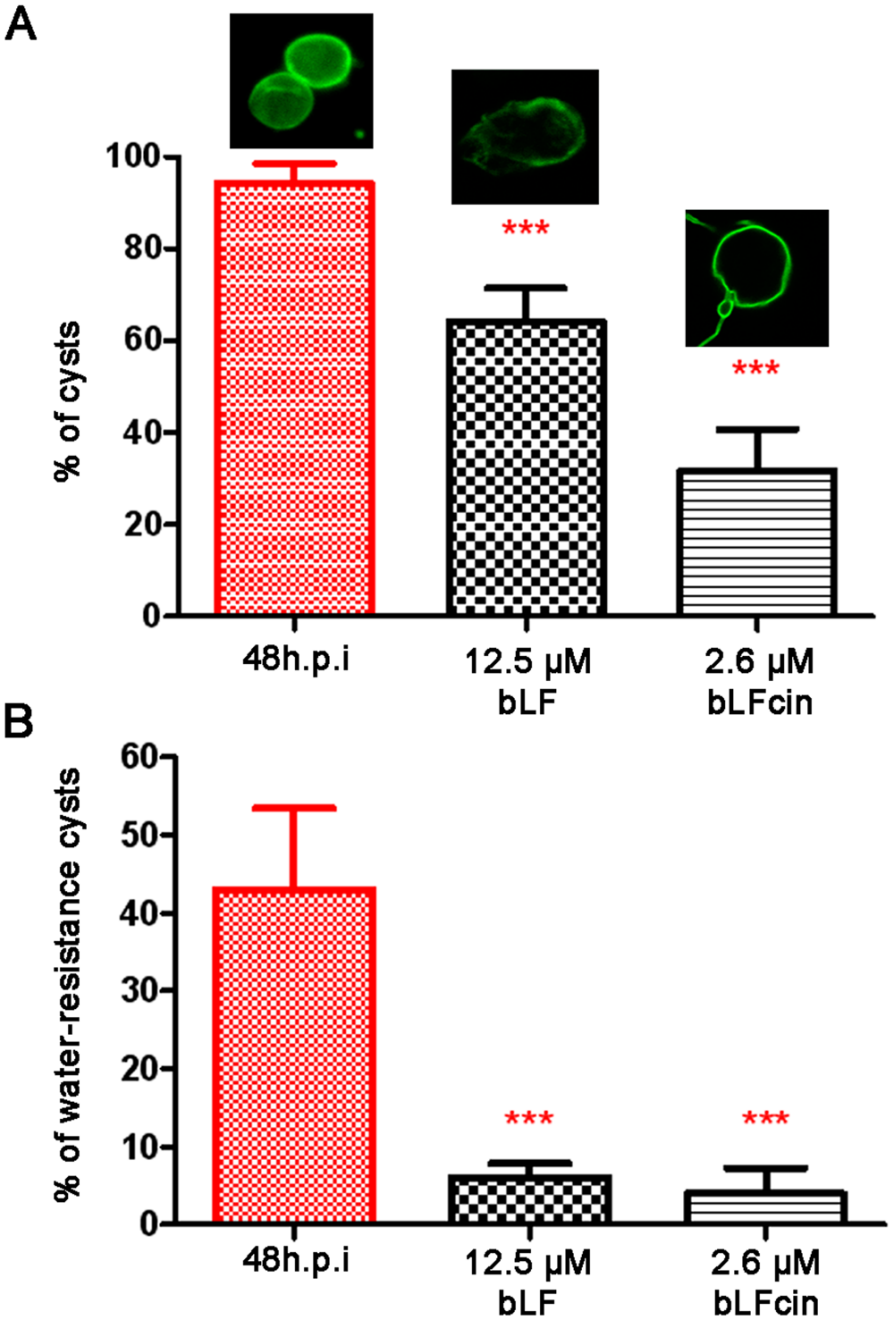
Treated trophozoites are unable to produce mature cysts. (**A**) Trophozoites exposed to 12.5 μM of bLF or 2.6 μM of bLFcin during encystation show a significant reduction in the percentage of cyst-like cells, compared with the non-treated control. (**B**) Bars show that the percentage of the cysts produced by cells treated with 12.5 μM of bLF or 2.6 μM of bLFcin is less than 8% water-resistant when compared with the control of non-treated encysting (48 h.p.i.) cells.

The fact that the expression of CWPs and formation of ESVs is necessary but not sufficient to produce mature-infective cysts, and no cysts were observed after adding low doses of bLF or bLFcin, prompted us to analyse whether the addition of bLF and bLFcin might favor the production of viable cysts when exposed to normal encystation conditions (encysting medium). We performed the *in vitro* high bile Uppsala encystation protocol^32^ to induce cyst production from trophozoites but now adding bLF or bLFcin, and used non-treated trophozoites as control. After 48 h of encystation, the number of cysts produced by treated cells was significantly lower than that of non-treated control cells (Fig. 8A). Moreover, treated cells produced unviable cysts-like cells, since these cysts were not water-resistant (Fig. 8B). Since the transmission of *G. lamblia* is primarily due to the presence of mature water-resistant and biologically active cysts in contaminated water supplies, the inhibition of trophozoite growth and the production of non-viable cysts by bLF and bLFcin make these natural molecules excellent candidates for controlling giardiasis.

## Discussion

This report showed that low concentrations of both bLF and bLFcin produced growth inhibition of *G. lamblia* trophozoites and triggered incomplete cell differentiation. Previous reports showed the killing effect of high concentrations of both molecules in *G. lamblia* trophozoites by causing severe morphological defects^19–21^, and suggested but did not demonstrate the implication of a receptor and the participation of the endocytosis machinery^19–21^. In *G. lamblia*, we previously reported that GlLRP participates in the binding and internalization of LDL and chylomicrons through a RME mechanism involving the adaptor proteins AP-2 and GlENTHp^23,24^. Here, we showed that, similar to what happened in other cells, bLF and bLFcin are internalized through the same pathway and that this mechanism is essential to trigger morphological changes and microstatic effects in treated trophozoites. This evidence also reinforces the findings that, in eukaryotic cells, the internalization of bLFcin depends on an energy-dependent pathway that is inhibited at 4 °C, and differs from the energy-independent mechanisms in which LFcin directly interacts with the plasma membrane producing membrane permeabilization and cell death in bacteria^33^. In this regard, Zumthor *et al*. 2017, showed the presence of static invaginations containing the giardial clathrin heavy chain that connect the space between the PM and the PV and suggested that they might function as unregulated points of extracellular material entry to the cell^34^. However, the role of these static structures is still unclear as was addressed by us in a previous publication^35^. It is possible that the clathrin heavy chain in this parasite also participates in the unregulated entry of material from the environment. Still, the critical experiment including depletion of clathrin to test for defects in endomembrane organization and RME has yet to be performed. There is extensive evidence that GlENTHp and the clathrin heavy chain are involved in the canonical RME machinery^23,24,36,37^. However, data from the group headed by Dr. Adrian Helh suggested that GlENTHp (named GlEpsin by this group) does not participate in RME because of its localization on the giardial ventral disk and the lack of evidence of GlEpsin interaction with endosomal components, including clathrin^26^. The differences between their data and others (including us) has been well addressed in several publications^24,35,38–40^. So far only LDL, chylomicrons and (now) bLF and bLFcin was proved to be endocytosed by RME, with the canonical machinery involved^23,24,36,37^.

More than 7000 articles describing the characterization and properties of LF from many origins have been posted in biomedical and life sciences journal databases. LF has been intensively studied as an iron-transferring molecule promoting iron absorption, mostly through its binding to specific LF receptors present in human mucosa cells^41–44^. Nevertheless, many other properties and particularities of LF have been discovered, such as its immunomodulatory, microbicidal, anti-inflammatory, and anti-carcinogenic activities^45^. LF can exist in nature in an iron-free form known as apoLF and as holoLF, with at least one of the two ferric ions attached. Although both types of lactoferrin interact with pathogenic microorganisms, apoLF can be microbicidal while holoLF can be used by bacteria, fungi and protozoa as a source of iron for growth^46–48^. One of the hypotheses was that the microbicidal effect of LF relied on its iron-binding capacity, withholding iron required by the microorganisms. However, the microbicidal action of LF on many microorganisms is stronger than iron deprivation itself and the addition of iron did not prevent killing^49–51^. Moreover, most of the bactericidal activity of hLF and bLF has been attributed to the iron-free N-terminal peptides of mature LF (human LFcin residues 1–47 and bLFcin residues 17–41), produced by pepsin cleavage of the native protein *in vitro* and probably *in vivo*^8,9,11^. Importantly, LFcin has more potent microbicidal activity *in vitro* than intact bLF (on a molar basis)^8,9,11^. In this context, it is clear that LF degradation may have beneficial protective functions rather than detrimental. However, as the degradation of LF depends on its source species, the temperature, the pH and the activity of enzymes, it is necessary to validate its action in different conditions to provide essential information.

Holo-human LF is endocytosed by its binding to the specific LfR present in the apical membrane of the intestinal cell and this uptake, together with the later iron release pathway, is part of the regulation of iron metabolism and iron content in the human body^44^. On the other hand, additional multifunctional properties of LF have been attributed to its binding to LRP receptors^52^. Unfortunately, the direct interaction of bLF and bLFcin with GlLRP was problematic since we were unable to successfully test this interaction by immunoprecipitation or pull-down using crosslinkers (Supplementary Fig. 2). However, few if any reports on LF showed direct interaction with a receptor but rather assessed their interaction indirectly, as we demonstrated in this report.

The effect of LF binding to LRP induces different signalling pathways depending on the target cells^53^. Thus, in the *Giardia* trophozoites treated with bLF and bLFcin, their interaction with GlLRP may be responsible for the signalling that induces the beginning of the encystation process. The participation of GlLRP in signalling has not yet been addressed but it may complete the already proposed mechanism of cholesterol-mediated regulation of encystation induction, in which the MAPK/ERK signalling pathway seems to be responsible^54^. Our results showing that low concentrations of bLF or bLFcin promoted the expression of CWP1, suggest that there may be a signal mechanism that triggers CWP expression. However, this signalling seems to differ with the one involved in triggering the encystation process since treated trophozoites were unable to differentiate into mature water-resistant cysts. This finding agrees with the data showing that not only the expression of CWPs but also the coordinated trafficking and deposition to form the cyst wall seems to be necessary to produce viable cyst^55–58^. Unexpectedly, the ER turned acid after bLF or bLFcin addition to trophozoites. The PVs and the PV-containing bare zone have been the only acidic organelles described in *G. lamblia*^23,59^. However, in the report of Ratner *et al*., 2008, although they stated that LysoTracker stained lysosomal vesicles (PVs), an image showed a typical ER pattern after addition of the plant lectin wheat germ agglutinin (WGA) to growing cells^60^. It is possible that the addition of WGA to growing trophozoites had a similar effect (ER stress??) than bLf or bLFcin. Since this experiment lacked the control in which cells without addition of WGA were stained with Lysotracker, a straight forward interpretation of the images would be inappropriate. We also found that trophozoites treated with low doses of bLF or bLFcin were still unable to differentiate into viable cyst when were placed in the encystation medium, suggesting that bLF and bLFcin produce an alteration of the differentiation process by un still unexplored mechanism. Several studies reported that changes in the pH, bile salts and cholesterol in the trophozoites’ environment are key requirements for the differentiation of trophozoites into water-resistant cysts^54,61^. It is well known that regulation of the internal pH of endomembrane compartments is required for their optimal function^62^. It is thus possible that the immature cysts could result from defects in the CWP trafficking due to disrupted endomembrane pH in bLf and bLfcin treated trophozoites. A detailed analysis of the intracellular pH variation during the parasite differentiation might help to understand the cellular homeostasis that occurs during encystation. A deeper analysis comparing the signalling, protein expression and trafficking between treated and untreated cells induced to encyst is necessary to disclose this matter. However, the fact that a low amount of bLF or bLFcin avoids the dissemination of the diseases by producing non-viable cyst is promising.

The unique characteristics of LF and its derived peptides provide an opportunity for treatment and nutritional improvements. It can be used as a supplement to increase iron absorption and free radical detoxification, since it was found that the iron from bLF was more easily absorbed across the intestinal mucosa than the iron from ferrous salts^63^. But, in addition to iron uptake, the benefits of its antimicrobial and regenerative activity give LF great therapeutic potential. In this scenario, our work supports the use of bLF to treat giardiasis. It may be beneficial in several aspects: at high doses it produces trophozoite killing, while traces (low concentration) cause microstatic effects associated with the production of cyst-like cells; LF protects the intestinal cell barrier^64^ and modulates the immune response^65,66^; and, finally, no side effects of the use of LF as a dietary supplement have been reported to date^67^. The current antigiardial therapy of choice is with nitroimidazoles, but the number of refractory cases, usually associated with drug resistance, suboptimal drug concentrations and immunocompromise, is increasing^68,69^. There are also frequent side effects associated with these drugs, including dizziness, trouble sleeping and severe gastrointestinal symptoms. It is clear that new potential pharmaceuticals from natural sources need to be evaluated to improve the chemotherapy treatment for giardiasis. The properties established for LF give it great therapeutic potential.

## Materials and Methods

### Antibodies and other reagents

Fluorescein isothiocyanate (FITC) conjugated anti-CWP1 monoclonal antibody (mAb) was purchased from Waterborne Inc. (New Orleans, LA, USA). 2F5 mAb was used for the μ2 subunit of AP-2^23^. Anti-VSP1267 5C1 mAb was a gift of Dr. Nash^70^. Anti-HA mAb, Apo-lactoferrin containing 4.1% iron, from bovine milk (bLF), poly-L-Lysine, formaldehyde, cysteine hydrochloride monohydrate, bovine serum albumin and low-density lipoprotein (LDL) from human plasma were purchased from Sigma-Aldrich (St. Louis, MO). FluorSave™ reagent was from Calbiochem (San Diego, CA). Human chylomicrons were acquired from Athens Research & Technology (Athens, GA, USA). LysoTracker™ Red DND-99 used to label PVs was acquired from Molecular Probes-Invitrogen (Carlsbad, CA). FITC-(N-Terminal) bLFcin (FKCRRWQWRMKKLGAPSITCVRRAF) was synthesized and labelled by GenScript® (Piscataway, NJ, USA). For FITC-Labeling bLF, 2 mg/mL of bLF was dissolved in 0.1 M sodium carbonate buffer pH 9 and incubated overnight with FITC (Sigma-Aldrich, St. Louis, MO). Then, 50 mM NH_4_Cl was added for 2 hours at 4 °C to stop the reaction. The unbound FITC was eliminated by dialysis using cellulose membranes (Sigma-Aldrich, St. Louis, MO) at 4 °C.

### *Giardia* cell lines

Standard cell lines: *Wild-type* trophozoites of the isolate WB, clone 1267^71^. Stable transfected cells: *lrp*_*-*_*ha*: puromycin resistant WB1267 overexpressing the HA C-terminus tagged GlLRP; *lrp:as* puromycin resistant WB1267 overexpressing antisense *lrp* RNA for GlLRP depletion^22^. *glenth-ha*: puromycin resistant WB1267 overexpressing the HA C-terminus tagged GlENTHp; *ds-glenth*: puromycin resistant WB1267 overexpressing Tetracycline-inducible *glenth* double stranded RNA (ds) for GlENTHp knock-down^24^. Trophozoites were cultured in TYI-S-33 medium supplemented with 10% adult bovine serum and 0.5 mg/mL bovine bile as described previously^72^.

### Growth curves

Growth curves were performed as previously described^23^. Briefly, the indicated cell lines were grown exponentially in 8-mL tubes with different μM concentrations of bLF or bLFcin (described in the corresponding figure legends) and cellular proliferation/viability measured at 6-, 12-, 24- and 48-h after treatment. The number of viable cells was determined by counting with a haemocytometer after addition of 0.4% Trypan blue, according to the instructions from the manufacturer. Results were analysed for statistical significance by two-way ANOVA with GRAPHPAD Prism 5 Data Analysis Software (GRAPHPAD Software Inc., La Jolla, CA, USA). Mean and standard mean values were calculated from at least three biologically and technically independent experiments. p < 0.001 was considered significant and was indicated by asterisks in the figures.

### Uptake experiments

The uptake assays were performed as reported^23^. Briefly, *wild-type*, *lrp-ha*, *lrp:as*, *glenth–ha* or *ds-glenth* trophozoites were incubated (or not, for control) with bLF or bLFcin in Labeling buffer (50 mM Glucose, 10 mM L-cysteine and 2 mM Ascorbic Acid in PBS, pH 7.2) at 4 °C, washed and placed at 37 °C for 30 min to allow the endocytosis. The trophozoites were then washed, fixed with 4% of formaldehyde and visualized by fluorescence microscopy. Fluorescence images were measured by the Fiji image processing package (http://fiji.sc/wiki/index.php/Fiji) over raw images of each cell. All images were acquired and processed identically. Competition binding assays were performed by growing *wild-type* cells in the presence of a 10-fold excess (100 μg/mL) of unlabelled LDL or 100 *μ*g/mL chylomicrons for 20 min in ice-cold Labeling buffer (0–4 °C). Then, the cells were washed and incubated with 25 µM FITC-bLF or 5.2 µM FITC-bLFcin first in ice-cold Labeling buffer, washed, and later maintained at 37 °C for 30 min for endocytosis. Cells were then harvested and fixed with 4% formaldehyde. All washes were performed twice by centrifugation at 600 × g with Labeling buffer at 4 °C. Preparations were mounted with FluorSave™ mounting medium and the fluorescence analysed with a motorized FV1000 Olympus confocal microscope (Olympus UK Ltd, UK), using 63x or 100x oil immersion objectives (NA 1.32).

### Extracellular protease digestion

5 × 10^5^ cells were incubated with 300 µg of Proteinase K (Productos Bio-Lógicos, IA09) and 600 µg of Trypsin from porcine pancreas (Sigma Chemical Co, USA) in buffer (50 mM Tris, 7.5 mM CaCl_2_, pH 8.8). Control trophozoites were incubated with the buffer without proteases. The cells were treated for 3 h at 37 °C with gentle mixing. Then, to inactivate the proteases, 20 µl of 0.1 M Phenylmethanesulfonyl fluoride (PMSF) was added. After treatment, 0.4% Trypan blue was added to corroborate plasma membrane integrity. The trophozoites were washed with PBS at 37 °C and incubated in Labeling buffer containing 12.5 µM of FITC-bLF, 2.6 µM of FITC-bLFcin, or 50 µg of FITC for 30 min at 37 °C. After washing the cells, they were attached to slides pretreated with poly-L-Lysine and fixed with 4% formaldehyde for 40 min at room temperature. Protease-treated and untreated control trophozoites were subjected to immunofluorescence assay as described below, using the anti-VSP1267 5C1 mAb without permeabilization or the anti-AP2 mAb 2F5 in fixed-Trito X-100 permeabilized cells.

### Immunofluorescence Assay (IFA)

IFA of fixed cells was performed basically as described^73^. Briefly, trophozoites or encysting cells were cultured in growth medium or encysting medium, respectively, harvested and allowed to attach to slides, pretreated with poly-L-lysine. After fixation with 4% formaldehyde in PBS, the cells were washed and blocked with 3% BSA (Sigma Chemical Co, USA) in 0.01% Saponin in PBS. Cells were then incubated with the primary Ab diluted in PBS containing 1.5% BSA and 0.01% Saponin at 37 °C, followed by incubation with Alexa 488 anti-mouse (dilution 1:500) secondary antibody at 37 °C. Finally, preparations were washed with PBS and mounted in FluorSave™ mounting medium. Fluorescence staining was visualized with a motorized FV1000 Olympus confocal microscope (Olympus UK Ltd, UK), using 63x or 100x oil immersion objectives (NA 1.32). Differential interference contrast images were collected simultaneously with the fluorescence images, by the use of a transmitted light detector. Images were processed using Fiji software^74^.

### LysoTracker Staining

The staining was performed as previously described^27^. Trophozoites, untreated or treated with 12.5 µM of bLF or 2.6 µM of bLFcin, were incubated at 37 °C for 30 min in growth medium containing 50 nM LysoTracker Red. After this treatment, the trophozoites were observed by video microscopy as described previously^30^.

### Image processing for stacks

For live cells stained with LysoTracker, stacked images were acquired at 10 f.p.s. Super-resolution radial fluctuations (SRRF) frames were produced by running the NanoJ-SRRF method, as described^75^.

### Transmission electron microscopy (TEM)

4 × 10^7^ *wild-type* trophozoites were incubated with 12.5 µM of bLF or 2.6 µM of bLFcin for 30 min at 37 °C. The trophozoites were washed twice with PBS at 37 °C and then fixed in a solution containing 2.5% glutaraldehyde, 4% paraformaldehyde, and sucrose 4% in 0.1 M cacodylate buffer. The cells were centrifuged for 5 min at 600 × g, washed in 0.1 M cacodylate buffer and postfixed for 60 min at room temperature in a solution containing 1% osmium tetroxide, 0.8% potassium ferrocyanide, and 5 mM CaCl_2_ in 0.1 M cacodylate buffer. Subsequently, the cells were washed in buffer, dehydrated in acetone and embedded in Polybed resin. Thin sections were stained with uranyl acetate and lead citrate and observed in a JEOL 1230 Transmission Electron Microscope.

### Scanning electron microscopy (SEM)

1 × 10^7^ *wild-type* trophozoites were incubated with 12.5 µM of bLF or 2.6 µM of bLFcin for 30 min at 37 °C. Samples were fixed and postfixed as described for TEM, dehydrated in ethanol series, dried by the critical point method in a Leica (EM CPD 030) apparatus, mounted on stubs, and covered with a 20 nm-thick gold layer in a Denton vacuum (desk IV) equipment. The samples were observed in a JSM-6390LV scanning electron microscope.

### bLF or bLFcin treatment and differentiation

Growing *wild-type* cells were incubated with increasing concentrations of bLF or bLFcin for 30 min at 37 °C in Labeling buffer (50 mM Glucose, 10 mM L-cysteine and 2 mM Ascorbic Acid in PBS, pH 7.2). After two 5 min wash of the cells with Labeling buffer alone (0–4 °C), the cells were harvested, fixed immediately with 4% formaldehyde and prepared for direct immunofluorescence as described above but using the FITC-labelled anti-CWP1 mAb. Control of the experiment included the mock-treatment of growing *wild-type* trophozoites to compare with the effect of bLF and bLFcin addition, and of *wild-type* trophozoites after 12 hour post-induction (h.p.i.) of encystation, as a control for IFA.

### Encystation of bLF and bLFcin treated trophozoites

5000 trophozoites/ml were induced to encyst with 12.5 µM of bLF or 2.6 µM of bLFcin (or without bLF or bLFcin for control of non-treated encysting cells). The same amount of growing cell were led to attach before addition of the encystation TYI-S33 medium, supplemented with 10% adult bovine serum and 2.5–5.0 mg/ml bovine bile at pH 7.8^32^.

### Water-treatment

Cysts and cyst-like cells obtained after bLF or bLFcin treatments were harvested and the remaining trophozoites lysed by incubation in double-distilled water for 48 h at 4 °C. Water-resistant cysts were washed 3 times and counted with a haemocytometer.

## Electronic supplementary material

Supplementary figure 1 to 3

## Data Availability

The datasets generated during and/or analysed during the current study are available from the corresponding author on reasonable request.
