# Supplementary material for: Lactoferrin and lactoferricin endocytosis halt Giardia cell growth and prevent infective cyst production
Source: Sci Rep. 2018 Dec 21;8:18020. doi: 10.1038/s41598-018-36563-1 (PMC6303297; doi:10.1038/s41598-018-36563-1)

**Lactoferrin and lactoferricin endocytosis halt *Giardia* cell growth and prevent infective cyst production**

Lorena S. Frontera[1], Sofía Moyano[1], Gonzalo Quassollo[1], Adriana Lanfredi-Rangel[2], Andrea S. Rópolo[1], and María C. Touz[1*]

[1]Instituto de Investigación Médica Mercedes y Martín Ferreyra, INIMEC – CONICET,

Universidad Nacional de Córdoba, Córdoba, 5000, Argentina.

[2]Serviço de Microscopia Eletrônica, Centro de Pesquisas Gonçalo Moniz, Fiocruz-Ba, 40296-710, Brazil

Running Head: Giardicidal action of lactoferrin and its N-peptide

*Corresponding author

Email: ctouz@immf.uncor.edu

**Supplementary Figure S1: GlLRP and GlENTHp are involved in the endocytosis of Bodipy-LDL.**

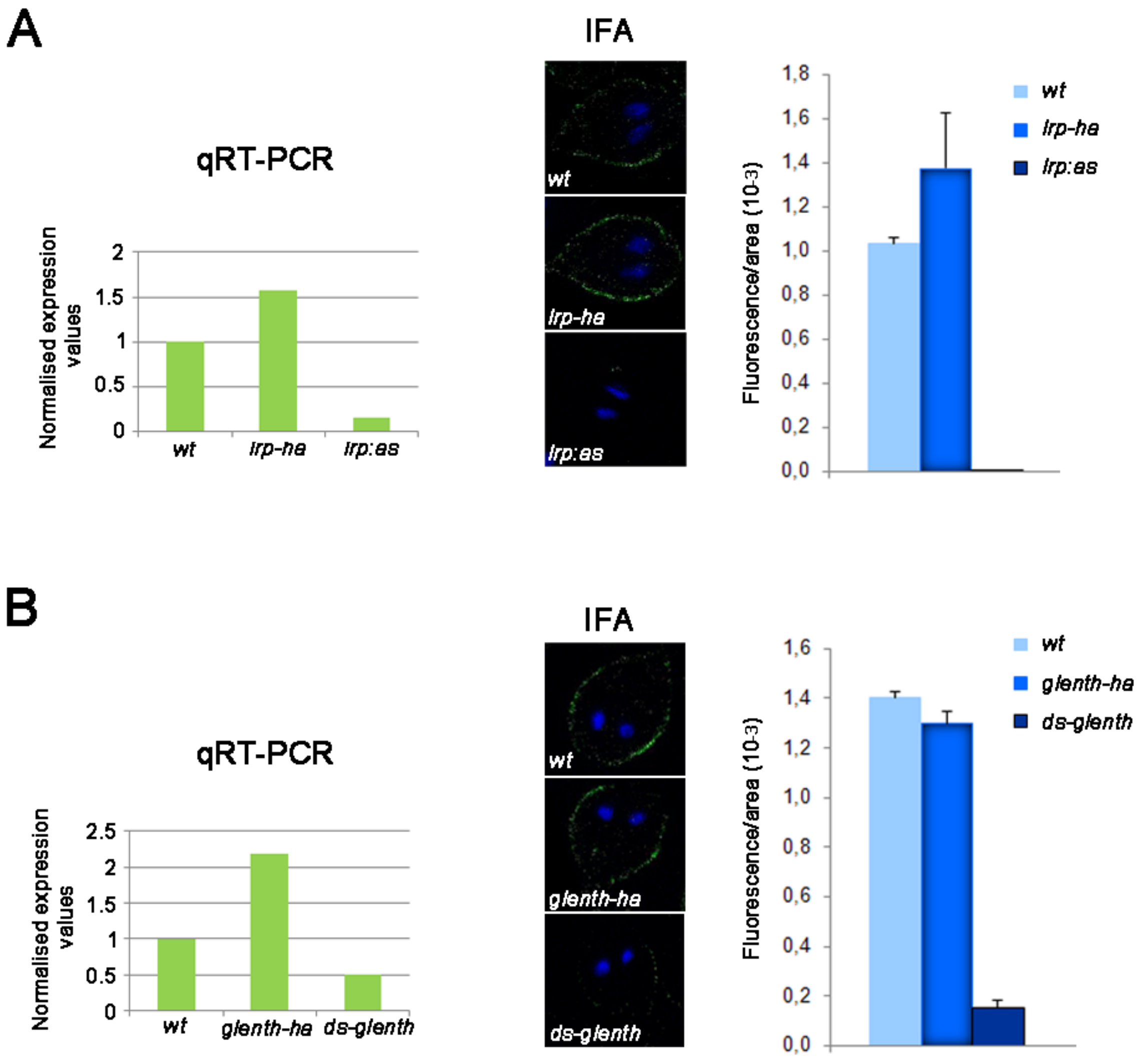


**GlLRP and GlENTHp are involved in the endocytosis of Bodipy-LDL.** (A) qRT-PCR: *lrp* gene expression in *wild-type* (wt), *lrp-ha*, and *lrp:as* trophozoites. Expression was normalized to the *gdh* housekeeping gene. Error bars indicate standard deviation for experiments with more than one trial. IFA: Confocal fluorescence microscopy of Bodipy-LDL endocytosis (up to 30 min) in *wild-type* (wt), *lrp-ha*, and *lrp:as* trophozoites. Nuclear DNA was labeled with DAPI (blue). Bars: Histograms of fluorescent images (fluorescence/area x $10^3$) show the relative amount of fluorescent Bodipy-LDL internalized by parasites in control (*wild-type*-wt-) and transgenic cells (*lrp–ha* and *lrp:as*).

(B) qRT-PCR: *enth* gene expression in *wild-type* (wt), *glenth-ha*, and *ds-glenth* trophozoites. Expression was normalized to the *gdh* housekeeping gene. Error bars indicate standard deviation for experiments with more than one trial. IFA: Confocal fluorescence microscopy of Bodipy-LDL endocytosis (up to 30 min) in *wild-type* (wt), *glenth-ha*, and *ds-glenth* trophozoites. Nuclear DNA was labeled with DAPI (blue). Bars: Histograms of fluorescent images (fluorescence/area x $10^3$) show the relative amount of fluorescent Bodipy-LDL internalized by parasites in control (*wild-type*-wt-) and transgenic cells (*glenth-ha* and *ds-glenth*).

**Supplementary Figure S2: LRP-HA - bLF immunoprecipitation and immunoblotting.**

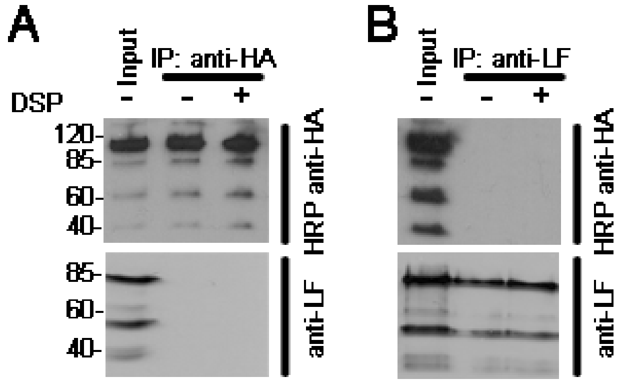


**LRP-HA - bLF immunoprecipitation (IPP) and immunoblotting:** (A) Whole-cell extracts were isolated from *lrp-ha* trophozoites exposed to bLF for 30 min. Immunoprecipitations of LRP-HA were carried out using anti-HA mouse mAb (SIGMA) and immunoblotted using HRP-labelled anti-HA mouse mAb (SIGMA) to detect LRP-HA or rabbit anti-LF polyclonal antibody (Fab Gennix) to detect bLF. (B) A reverse immunoprecipitation assay was conducted from *lrp-ha* trophozoites exposed to bLF for 30 min using the rabbit anti-LF polyclonal antibody and immunoblotted using the rabbit anti-LF polyclonal antibody to detect bLF or the HRP-labelled anti-HA mouse mAb to detect LRP-HA. IPP was performed as described by Krtková et al. 2017, using the DSP cross-linker. All experiments were repeated three times with different sets of samples and nonspecific IgGs were used as a negative control for immunoprecipitation. Input: *lrp-ha* trophozoites extracts exposed to bLF for 30 min.

Krtkova, J. *et al.* 14-3-3 Regulates Actin Filament Formation in the Deep-Branching Eukaryote Giardia lamblia. *mSphere* **2**, doi:10.1128/mSphere.00248-17 (2017).

**Figure S3. Full Western blot scans for Supplementary Fig. 2.**
**The full scans for each blot are shown on the bottom panels.**

**Each sample is explained in the Fig. S2 and denoted with the letters a to i.**

Fig S2

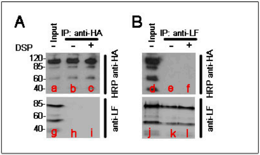


Full WB scans

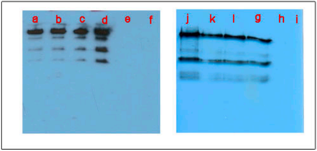

Supplement: Supplementary file 1 — Supplementary figure 1 to 3 [file 41598_2018_36563_MOESM1_ESM.pdf]
